# Establishment and Evaluation of Fatigue Mice Model in the Convalescence Phase of Influenza A

**DOI:** 10.3390/v17050593

**Published:** 2025-04-22

**Authors:** Xiaoke Zeng, Cheng Zhang, Jianing Shi, Xuan Ji, Keying Wang, Ling Li, Qinghu He

**Affiliations:** 1 College of Traditional Chinese Medicine, Hunan University of Chinese Medicine, Xueshi Road Campus, Changsha 410208, China; asteria@stu.hnucm.edu.cn (X.Z.); 20233665@stu.hnucm.edu.cn (C.Z.); 20233606@stu.hnucm.edu.cn (J.S.); 20243610@stu.hnucm.edu.cn (X.J.); 202102150250@stu.hnucm.edu.cn (K.W.); 2 School of Rehabilitation Medicine and Health Care, Hunan University of Medicine, Jinxi Road Campus, Huaihua 418000, China

**Keywords:** influenza A, convalescence phase, animal model, fatigue, pneumonia, suboptimal health status

## Abstract

Certain strains of Influenza A virus (IAV), a primary cause of influenza, can lead to pneumonia. Patients recovering from influenza pneumonia may experience physical discomfort akin to post-acute sequelae of COVID-19 (PASC). Despite extensive clinical research on viral pneumonia during convalescence, animal model studies are scarce, highlighting the need for a reliable model for pharmaceutical research. In this study, BALB/c mice were divided into three groups: NC (control), MC (infected with IAV), and Model (treated with oseltamivir post-infection for five days). A fatigue model was then induced in the Model group through diet restriction and weight-bearing swimming. The results showed the MC group had a 75% survival rate, while the NC and Model groups had 100%. Both the MC and Model groups experienced rapid weight loss followed by gradual recovery, differing significantly from the NC group. From dpi (days post-inoculation) 6 to dpi9, the MC group lost more weight than the NC group. The MC group had the highest pulmonary index, but there was no significant difference in *IAV Nucleoprotein (NP)* expression across groups. The Model group had higher *IL-10* levels than the NC and MC groups, while the MC group had the highest *TNF-α* expression. Hematoxylin and eosin (H&E) staining revealed pathological changes in the MC and Model groups, with severe damage and pulmonary fibrosis in the MC group. Oxidative stress markers showed the MC group had the highest lactate dehydrogenase (LDH) and malondialdehyde (MDA) levels and lowest superoxide dismutase (SOD) activity. Electron microscopy indicated mitochondrial damage in both the MC and Model groups. The Model group had the lowest splenic and thymic indices, with histological findings showing larger splenic nodules in the MC group and poor thymocyte density and atrophy in the Model group. The successful creation of this mouse model of influenza pneumonia convalescence phase fatigue, exhibiting fatigue syndrome with various symptoms, holds significance for PASC and other viral pneumonia convalescence phase animal model research.

## 1. Introduction

Influenza A is the most prevalent influenza, caused by IAV [1] and transmitted mostly by droplets and other ways. In the spring of 2023, the H1N1 pandemic influenza resurfaced in China, posing a hazardous situation by overlapping with the risk of COVID-19 infection, which has received broad social attention. Seasonal influenza primarily affects the upper respiratory tract and is generally self-limiting [2], whereas pandemic strains of the influenza virus and occasional seasonal strains can affect the lower respiratory tract, resulting in influenza pneumonia [3] and possibly inducing a cytokine storm in the body [4]. Current influenza prevention and treatment strategies emphasize vaccination and the use of antiviral medicines. However, in addition to preventing and treating influenza virus infection, the convalescence phase of H1N1 pneumonia requires care and effort. Animal studies and clinical research during the convalescence phase of influenza virus infection have shown [5,6,7] that after the resolution of H1N1 lung infection, it can still have a negative impact on the body for 3 to 4 weeks or even longer, including fatigue, decreased ability to work and move, decreased immunity, changes in pulmonary imaging, and even the induction of other diseases such as heart disease, which is similar to the post-COVID-19 condition [8].

There are numerous clinical studies on the convalescence phase of viral pneumonia, including H1N1 and COVID-19, but there are fewer studies on animal models, which is not conducive to drug development, and there is an urgent need to establish a stable animal model. Our exploratory studies on the post-influenza convalescence phase of mouse models were initiated in spring 2023, with the literature revealing that fatigue represents a predominant clinical manifestation during the convalescent phase of influenza-associated pneumonia [9]. According to traditional Chinese medicine (TCM) theory, Qi-deficiency, fatigue, and decreased immunity during the recovery period of influenza are consistent with the syndrome of damaged vital energy and remaining pathogenic factors, which is widely recognized in TCM [10,11]. The abnormal state of the body during this stage is analogous to post-viral fatigue syndrome (PVFS) [12], which is classified as a sub-health state [13] or suboptimal health status (SHS) [14,15] in modern medicine. As a result, the modeling method used animal models of Qi deficiency [16,17] and sub-health-status animal models [18,19,20]. The model was created by combining the low expression of *IAV NP* during the convalescence phase of influenza pneumonia with the organism’s steady increase in body weight. Male BALB/c mice, which are relatively stable for H1N1 virus infection [21,22], were chosen as the model animals.

## 2. Materials and Methods

### 2.1. Animals

A total of 36 specific pathogen-free (SPF) male BALB/c mice, aged 6–8 weeks, were obtained from Hunan SJA Laboratory Animal Co., Ltd., Changsha, China (Animal Quality Certificate No. 430727231100325042). The mice were housed in the animal experimental center at Hunan University of Chinese Medicine under permit No. SYXK (Hunan) 2019-0009. Each cage accommodated 4 to 5 mice, and the environmental conditions were maintained with a room temperature of 22–26 °C, a relative humidity of 45–50%, and a 12 h:12 h light/dark cycle, with free access to water. The experimental protocol was approved by the Animal Ethics Committee of Hunan University of Chinese Medicine (Approval No. LL2023022206).

### 2.2. Virus Strain

The influenza virus mouse lung-adapted strain (Type A, IAV, A/PR/8/34) was provided by the Virology Research Laboratory at Hunan Normal University and passed via the allantoic cavity of 10-day-old embryonated chicken eggs. The trials involved viruses with a hemagglutination titer of 1:640 or greater. The viral allantoic fluid was diluted with sterile physiological saline to a concentration of 50LD_50_ per 0.1 mL and stored at −80 °C for later use.

### 2.3. Drugs and Reagents

To prepare the suspension, 25 mg of (calculated as oseltamivir) phosphate granules were fully dissolved in distilled water and stored at −4 °C in a refrigerator. The commercial reagents and materials used in this study included oseltamivir phosphate granules (Yichang East Sunshine Yangtze River Pharmaceutical Co., Ltd., Yichang, China; Cat# 582210005), TRIzol Reagent (Simgen Biotech Co., Ltd., Hangzhou, China; Cat# 5301100), a reverse transcription kit (Novoprotein Technology Co., Ltd., Shanghai, China; Cat# E047-01B), an RT-qPCR kit (Novoprotein Technology Co., Ltd., Shanghai, China; Cat# E096-01B), 4% paraformaldehyde (Biosharp Biological Co., Ltd., Hefei, China; Cat# BL539A), phosphate-buffered saline (PBS; Procell Life Science & Technology Co., Ltd., Wuhan, China; Cat# WH0021D081), a lactate dehydrogenase (LDH) colorimetric assay kit (WST-8 method; Elabscience Biotechnology Co., Ltd., Wuhan, China; Cat# E-BC-K766-M), a malondialdehyde (MDA) assay kit (Elabscience Biotechnology Co., Ltd., Wuhan, China; Cat# E-BC-K318-M), a total superoxide dismutase (T-SOD) colorimetric assay kit (WST-1 method; Elabscience Biotechnology Co., Ltd., Wuhan, China; Cat# E-BC-K020-M), laboratory-grade pure water (Wuhan Servicebio Technology Co., Ltd., Wuhan, China; Cat# G4701), 2.5% glutaraldehyde fixative solution (Wuhan Servicebio Technology Co., Ltd., Wuhan, China; Cat# G1102-1.5ML), absolute ethanol (Tianjin Hengxing Chemical Preparation Co., Ltd., Tianjin, China; CAS No. 64-17-5), xylene (China National Pharmaceutical Group Co., Ltd., Beijing, China; Cat# 10023418), and a hematoxylin and eosin staining kit (Beyotime Biotechnology Co., Ltd., Shanghai, China; Cat# C0105S). All the RT-qPCR primer sequences were produced by Sangon Biotech Technology Co., Ltd., Shanghai, China, and particular sequences are listed in Table 1.

### 2.4. Experimental Methods

#### 2.4.1. Experimental Grouping and Model Establishment

The experiment included three groups: a negative control (NC) group, a model control (MC) group, and a model (Model) group, each with 10 mice. The experimental animals were kept adaptively for 5 days before being weighed.

**Figure 1:**
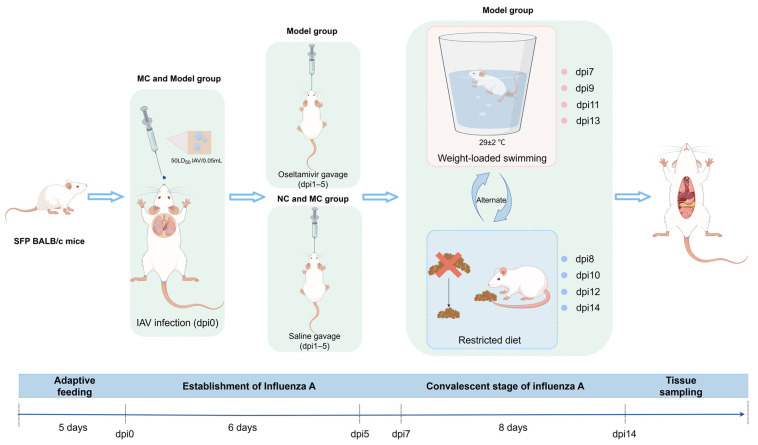
Establishment process of a fatigue model in mice during the convalescence phase of influenza A (By Figdraw 2.0).

Mice in the NC group were raised under normal conditions without intervention. Mice in the MC and Model groups were inoculated with IAV by uniformly dripping 0.05 mL of a 50LD50 influenza virus solution into each nostril, according to the project team’s earlier research [23]. The Model group was given oseltamivir within 24 h post-viral inoculation. Oseltamivir was administered via oral gavage at a therapeutically equivalent dose (determined using the animal’s body mass ratio per kilogram to the human body surface area) for 5 days in a row. The dose was 0.2275 mg of oseltamivir per 10 g of body weight per day, with the NC and MC groups receiving an equal volume of physiological saline. Following oseltamivir administration, the Model group was given an alternating regimen of limited nutrition and weight-bearing exhaustive swimming to replicate the symptoms of influenza A pneumonia convalescence (Figure 1). The specific protocol was as follows: Starting from dpi7, on non-dietary restriction days (i.e., negative geotaxis exhaustive swimming days), food supply was not limited. On dietary restriction days, the 24 h food consumption of the mice was precisely measured by weighing, with subsequent food provision restricted to 50% of this quantified amount. On negative geotaxis exhaustive swimming days, each mouse was placed in an opaque cylindrical container of water with a temperature of 29 ± 2 °C for weight-bearing swimming (with a weight tied to the base of the tail equivalent to 10% of the mouse’s body weight) until exhaustion (defined as the mouse’s nose being submerged for 3 to 5 s or signs of limb movement disorder). Mice in both the NC and MC groups received adaptive swimming training over the same time period. Following swimming, all mice were immediately placed in a warming cage with a heater to dry off. This procedure was repeated once every day for seven days in a row.

#### 2.4.2. Body Weight Monitoring

Starting from the first day of modeling, the body weight of all the mice was monitored daily using a high-precision electronic balance (accuracy ± 0.01 g) at a fixed time period each day throughout the experiment’s duration. For each mouse, the daily percentage of body weight loss relative to the initial body weight recorded on day 1 and the maximum weight reduction were calculated and recorded. All the mice sacrificed during the modeling procedures were rigorously excluded from the body weight data collection to ensure analytical integrity and prevent confounding variables.

#### 2.4.3. Syndrome Observation

Changes in fur condition, posture anomalies, hunched back, responsiveness, activity status, dietary behaviors, and eye squinting of mice in each group following viral inoculation and during the model establishment procedure were observed and documented.

#### 2.4.4. Organ Index

After the mice were weighed and euthanized, the thoracic and abdominal chambers were opened to remove the intact lungs, spleen, and thymus. The organs were weighed separately on an automated analytical balance. The spleen index, pulmonary index, and thymus index were determined using the following formula: organ index = (weight of the organ/body weight of the animal) × 100%.

#### 2.4.5. Final Exhaustive Swimming Duration

Following the model establishment (dpi14), all the groups of mice underwent a final exhaustive swimming test. A lead wire equivalent to 10% of each mouse’s body weight was secured at the base of its tail. Mice were individually placed in opaque cylindrical containers filled with water (25 ± 1 °C) and forced to swim until exhaustion, defined as the point when the mouse’s nose remained submerged for more than 5 s. Mice meeting this criterion were immediately removed. To prevent interference from physical contact between animals, only one mouse was tested per container at a time. Exhaustion duration was recorded in minutes (min). During the data analysis, outliers caused by operational errors (e.g., detachment of lead weights) were excluded to ensure result validity.

#### 2.4.6. Histopathological Observation

Following tissue collection, lung, spleen, and thymus specimens were fixed in 4% paraformaldehyde for 72 h, dehydrated through a graded ethanol series (50% to 100%), and embedded in paraffin for sectioning into 4 μm thick continuous slices. For the hematoxylin and eosin (H&E) staining, sections were sequentially processed as follows: stained with hematoxylin for 5 min, differentiated in 1% acid ethanol, counterstained with eosin for 2 min, and finally mounted with neutral resin. Tissue morphology was examined under a light microscope using a 10× objective to evaluate global architecture (e.g., alveolar integrity in lung tissue, follicular distribution in spleen) and a 40× objective to assess cellular details (e.g., inflammatory cell infiltration, hyperplasia in splenic red pulp).

#### 2.4.7. Virus Expression Level Determination

Approximately 50 mg of lung tissue (equivalent to the size of a soybean) was rapidly frozen in a liquid nitrogen-precooled mortar and pulverized into powder. The homogenized tissue was lysed with TRIzol reagent, and total RNA was isolated using a commercial RNA extraction kit. RNA concentration and purity were determined spectrophotometrically (A260/A280 ratio). Subsequently, RNA was reverse-transcribed into cDNA using a reverse transcription kit.

For real-time quantitative PCR (qPCR), specific primers targeting *IAV NP* and the reference gene *GAPDH* were designed. The reaction mixture contained cDNA template, primer pairs (0.125 μM final concentration), and SYBR Green supermix. Amplification was performed under the following thermal cycling conditions: 95 °C for 10 min (initial denaturation), followed by 40 cycles at 95 °C for 15 s and 60 °C for 1 min. Post-amplification melting curve analysis (65–95 °C, 0.5 °C increments) confirmed product specificity, with all the samples exhibiting single-peak dissociation curves. Relative gene expression was calculated using the 2^−ΔΔCt^ method.

All the procedures were performed on ice to prevent RNA degradation. Three technical replicates were included for each sample to ensure data reproducibility.

#### 2.4.8. Tissue Pro-Inflammatory and Anti-Inflammatory Cytokine Expression Levels

The operation procedures were the same as in Section 2.4.7, with primers for *IL-10*, *IL-6*, *TNF-α*, and *TGF-β* added. During the detection step, unimodal melt curve characteristics were confirmed as a quality control criterion for specific target amplification.

#### 2.4.9. Oxidative Stress Indicators Detection

Fresh peripheral blood collected from mice was allowed to sediment at room temperature for 30 min, followed by centrifugation at 3000 rpm for 15 min to isolate the serum. T-SOD, MDA, and LDH Assay Kits were used to measure the activity of SOD, MDA, and LDH in the mouse serum.

#### 2.4.10. Electron Microscopy Observation of Mitochondria in Cells

Fresh tissue from the mice was rinsed in 4 °C PBS to remove surface blood, blotted dry with sterile filter paper, and cut into <1 mm^3^ pieces to minimize mechanical damage. The tissue blocks were immediately fixed in pre-cooled 2.5% glutaraldehyde (0.1 M phosphate buffer, pH 7.4) at 4 °C for 2 h, followed by three 15 min washes in phosphate buffer. Secondary fixation was performed in 1% osmium tetroxide (0.1 M phosphate buffer) for 2 h in the dark. After dehydration through a graded ethanol series (50%, 70%, 80%, 90%, and 100%, 15 min each at 4 °C) and acetone substitution, the samples were infiltrated with acetone–epoxy resin mixtures (2:1 for 4 h; 1:2 overnight) and pure resin (37 °C, 3 h), then polymerized at 60 °C for 48 h. Ultrathin sections (50–70 nm) were cut using an ultramicrotome, mounted on copper grids, and double-stained with 3% uranyl acetate (30 min) and lead citrate (10 min). Mitochondrial ultrastructure was examined under a transmission electron microscope. Critical quality controls included maintaining 4 °C during processing to avoid swelling and ensuring section thickness of 50–70 nm for optimal resolution.

### 2.5. Statistical Methods

The statistical analysis was carried out using GraphPad Prism 9.5.0. The Shapiro–Wilk test was performed to assess normality, and the Kruskal–Wallis H test was utilized when the data did not fit a normal distribution. When the data met the assumption of variance homogeneity, multiple group comparisons were performed using one-way ANOVA at α = 0.05. If the variances were not homogeneous, Welch’s ANOVA was applied. When the data included two categorical factors, two-way ANOVA was used.

## 3. Results

### 3.1. Syndrome Observation in TCM

Mice in the NC group fed and drank normally, had smooth and lustrous hair, no abnormal posture or mental state, were agile and active with normal excreta, and showed no evidence of eye squinting or curling up. After dpi3, mice in the MC and Model groups demonstrated decreased activity, lethargy, disordered and dull hair, and soft stool texture. Hunched backs, eye squinting, and obvious indentations on both sides of the abdomen were also common in the MC group mice, with the most severe abnormalities occurring between dpi6 and dpi8, including MC mouse deaths. From dpi10 to dpi12, some mice in the MC group and all animals in the Model group started to improve in their symptoms.

### 3.2. Body Weight Changes and Survival Rates

**Figure 2:**
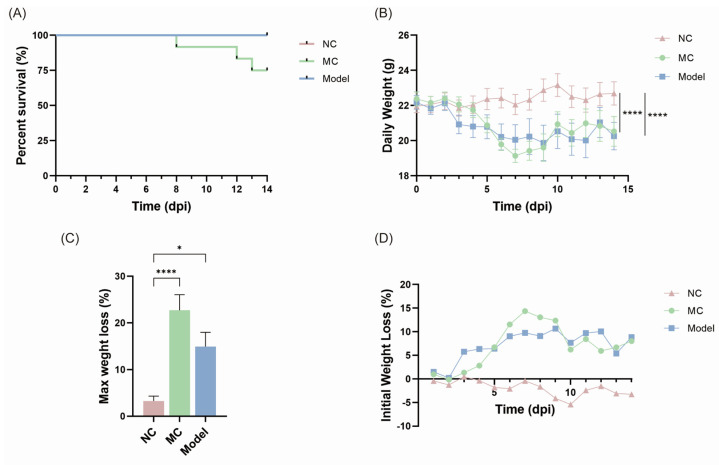
A comprehensive assessment of viral load, body weight changes, and mortality in mice. Percentage of survival from dpi0 to dpi14 in the MC and Model groups (**A**); daily weight changes in all groups of surviving mice (**B**); maximum weight loss (**C**) and initial weight loss (**D**) of mice in all groups. In Panel A, the line segment of the NC group is obscured by that of the Model group, with the two lines exactly coinciding. * *p* < 0.05, **** *p* < 0.0001.

Figure 2A depicts the survival rates of mice in the MC and Model groups on dpi14, with all animals in the NC and Model groups surviving, while one mouse in the MC group died on dpi8, dpi12, and dpi13, respectively, for a final survival rate of 75%. The analysis of daily body weight changes in all the surviving mice (Figure 2B) revealed that the body weight of the NC group mice progressively increased, but the body weight of the MC and Model group animals rapidly decreased in the first 7 days and subsequently slowly increased. A comparison of average body weight across the three groups revealed substantial differences between the MC and Model groups relative to the NC group (**** *p* < 0.0001), but no significant difference between the MC and Model groups (*p* = 0.8538).

Two-way ANOVA was used to compare the daily body weights of MC and Model group mice to those of NC group mice, and the results showed that the body weight difference between MC and NC group mice became more significant from dpi6 to dpi9, whereas no statistical difference was found when Model group mice were compared to NC group mice.

In Figure 2C, the MC group mice lost the most weight compared to the NC group (**** *p* < 0.0001). The Model group also lost weight compared to the NC group, but the difference was less significant (* *p* < 0.05). In terms of initial weight reduction percentages (Figure 2D), dpi6 to dpi9 were the four time points with the largest weight loss in the MC group.

### 3.3. Pneumonia Convalescence Phase of Mice

**Figure 3:**
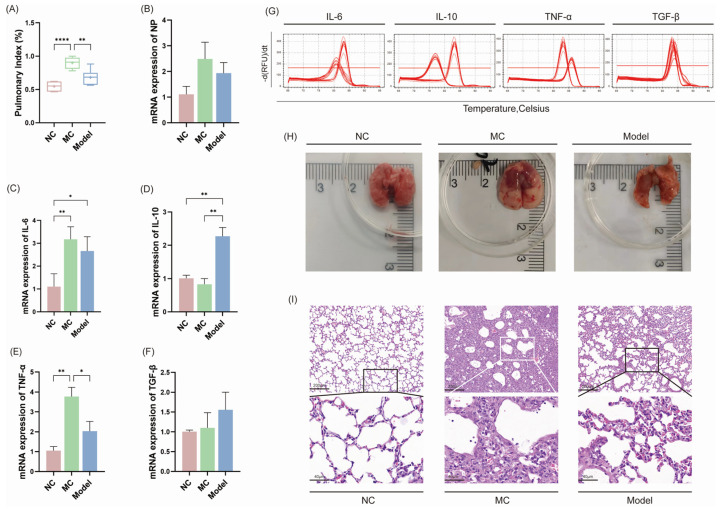
Recovery of lung inflammation in mice. Pulmonary index for all groups (**A**); pulmonary mRNA expression of IAV NP (**B**); mRNA expression of cytokines *IL-6*, *IL-10*, *TNF-α*, and *TGF-β* in mouse lung tissue (**C**–**F**); melting curve peak of *IL-6*, *IL-10*, *TNF-α*, and *TGF-β* (**G**); photograph of mouse lung tissue (**H**); field of H&E stained lung tissue sections at 10× and 40× magnification under a microscope (**I**); * *p* < 0.05, ** *p* < 0.01, **** *p* < 0.0001.

Figure 3A shows that the MC group had a significantly higher pulmonary index than the NC group (**** *p* < 0.0001) and the Model group (* *p* < 0.05). However, there were no significant variations in *IAV NP* expression levels between the three groups (Figure 3B), and they were low, indicating negative viral expression.

Regarding cytokine expression levels (Figure 3C–F), the Model group expressed considerably more *IL-10* than the NC and MC groups. The MC group had the highest expression of the inflammatory cytokines *TNF-α* and *IL-6* when compared to the other two groups, while this decreased in the Model group compared with the MC Group.

The photograph of the mouse lung tissue results (Figure 3H) showed that the lung tissue from the NC group mice appeared pink, with a smooth and shining surface, soft texture, intact lobes, and no visible nodules or masses. The lung tissue from the MC group was grayish-white in color, hard and full in texture, and brilliant red in the center. The Model group showed mild tissue atrophy and pulmonary edema.

The H&E results for the lung tissue (Figure 3I) revealed that the NC group mice had clear and intact alveolar structures with alveolar walls, no edema, and no neutrophil or macrophage infiltration. The MC group had fused alveolar septa, thickened alveolar walls, compensatory dilatation of some alveolar gaps, and high macrophage infiltration in the alveolar interstitium. The Model group had partially destroyed alveolar walls that fused with neighboring alveolar walls, limited neutrophil and macrophage infiltration, no substantial exudate, and largely intact lung tissue structure.

### 3.4. Fatigue-Related Behavioral Experiments and Laboratory Indicators

Mice in the NC group had a significantly longer period of loaded swimming to exhaustion compared to the MC and Model groups (*** *p* < 0.001, **** *p* < 0.0001), but there was no significant difference between the MC and Model groups.

**Figure 4:**
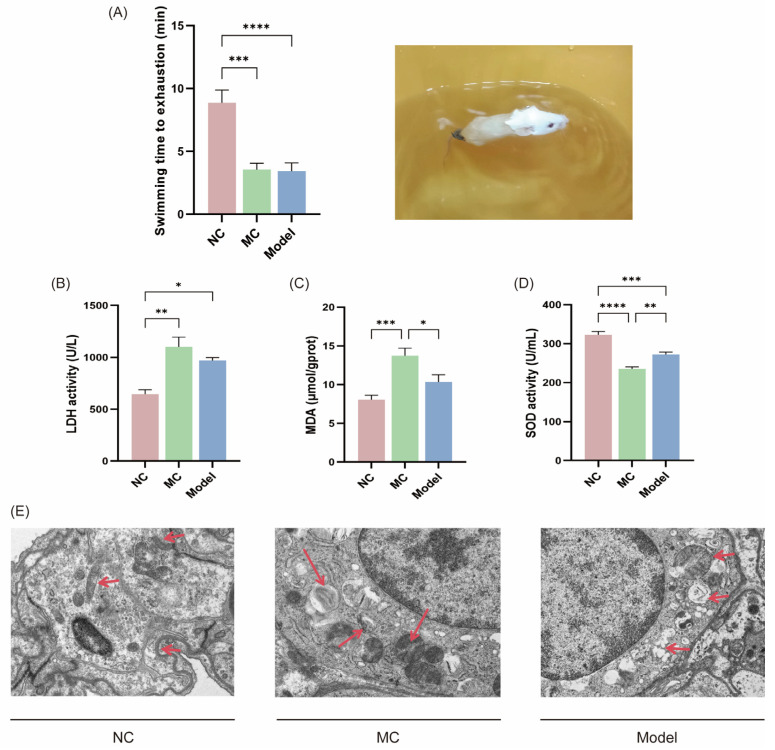
Fatigue-related behavioral studies and laboratory parameters. The duration of exhaustion during weight-bearing swimming (**A**); SOD activity (**D**), MDA and LDH levels (**B**,**C**); mouse tissue electron microscopy evidence at a magnification of ×10 k (**E**). In the electron microscopy results, the red arrows in the NC group from top to bottom highlight irregularly shaped mitochondria, intact mitochondrial membrane structures with distinct cristae arrangement, and tight junctions of the cell membrane. In the MC group, the red arrows from left to right indicate autolysosomes, autophagosomes, and swollen mitochondria. In the Model group, the red arrows from top to bottom demonstrate swollen mitochondria, autophagosomes, and cytoplasmic vacuoles within the cytoplasm. * *p* < 0.05, ** *p* < 0.01, *** *p* < 0.001, **** *p* < 0.0001.

In the three serum oxidative stress indicators (Figure 4B–D), the MC group exhibited the highest levels of LDH and MDA, followed by the Model group. The trend of SOD activity was inversely associated with LDH and MDA levels, with the lowest activity observed in the MC group and highest levels in the NC group, correlating the findings across all three indices.

Transmission electron microscopy analysis demonstrated that the NC group maintained normal mitochondrial morphology with intact cellular membranes and optimal cellular integrity. In contrast, the MC group exhibited characteristic autolysosomes, autophagosomes, and mitochondrial swelling, indicative of augmented autophagic activity. The Model group similarly displayed sustained mitochondrial edema and persistent autophagic vacuoles. Both the MC and Model groups showed ultrastructural evidence of mitochondrial damage.

### 3.5. Immune Organ Indices and Histopathological Assessment

**Figure 5:**
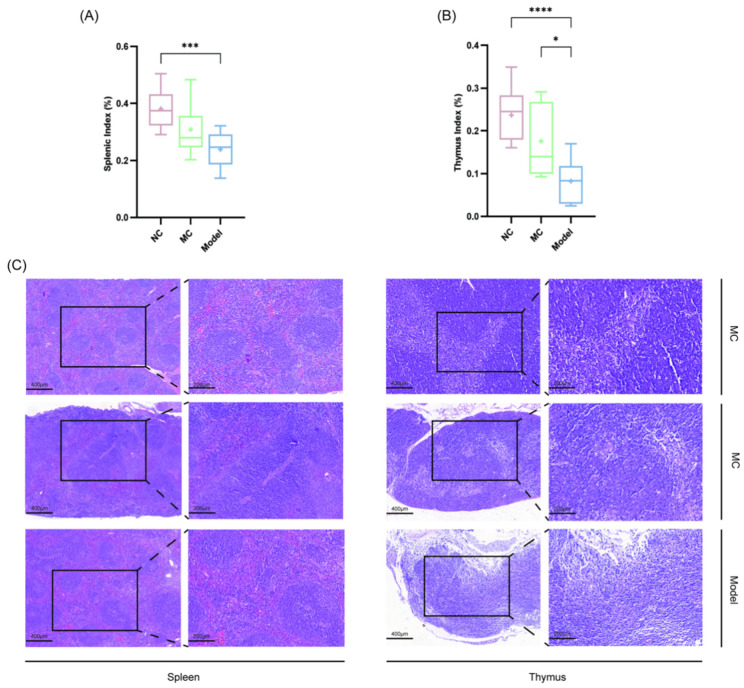
Immune organ indices and histopathological assessment. Mouse spleen organ index (**A**) and thymus organ index (**B**); H&E section field results of spleen and thymus tissues at 10× and 20× magnification (**C**); * *p* < 0.05, *** *p* < 0.001, **** *p* < 0.0001.

Among the three groups of mice, the Model group had the lowest spleen index (Figure 5A) and thymus index (Figure 5B), with significant differences compared to the other groups (** *p* < 0.05; *** *p* < 0.001; **** *p* < 0.0001).

Figure 5C shows the H&E staining results for the spleen and thymus tissues. In the NC group, the spleen tissue exhibited distinct white pulp and red pulp areas, with well-defined splenic nodules appearing as clearly circumscribed round structures. The splenic architecture was clear, with an even distribution of cells and no visible evidence of pathogenic infiltration or destruction. Splenic nodules in the MC group expanded and merged, leaving ambiguous borders. The Model group had more and larger splenic nodules.

The thymus cortex and medulla were well defined in the NC group, with apparent thymic corpuscles and adipose cells. The MC group had unclear thymic corpuscles, a smaller thymic area than the NC group, and symptoms of atrophy. The Model group had a low density of thymocytes in the medulla, significant thymic atrophy, and fuzzy boundaries between the cortex and medulla.

## 4. Discussion

A comprehensive review of the data reveals that the Model group mice have a lower mortality rate than the MC group, with a more consistent pattern in body weight changes. Fatigue modeling keeps the body weight of the Model group mice at a lower level after recovery, which is advantageous to the model. The Model group mice recovered from pneumonia, while the MC group retained inflammation and lung fibrosis. Compared to the NC and MC groups, the Model group mice exhibited considerable tiredness and oxidative stress, with conditions similar to the MC group.

During the model establishment process, the lung tissues of mice in the convalescence phase of influenza pneumonia are prone to fibrosis, which leads to impaired pulmonary ventilation function. Administering oseltamivir in the early stage of viral infection can avoid severe lung damage or high mortality caused by the superimposition of IAV infection and fatigue modeling during the modeling process [24]. This ensures the reproducibility of the experiment and the stability of the animal model and reduces experimental errors.

Combining the body weight and changes of the Model group mice with the modeling process, it can be found that starting from dpi8, the body weight measurement results after each 24 h restricted diet showed significant decreases compared to the previous day. This is because a restricted diet induces metabolic adaptation by reducing energy intake, leading to a decline in basal metabolic rate [25]. This metabolic adaptation may make it difficult for mice to rebuild energy reserves during the recovery period [26] and may even cause impaired immune function. Weight-loaded exhaustive swimming or forced swimming has been proven in previous studies to reduce organ indices of two major immune organs in mice: the spleen [27] and thymus [28], which aligns with the findings of this study.

Under the conditions of tissue damage, inflammation, and oxidative stress, serum LDH and MDA levels increase. Although the use of oseltamivir in the Model group can alleviate inflammation and oxidative stress, thereby reducing cellular damage caused by H1N1 pneumonia, both weight-loaded exhaustive swimming experiments and viral infection can elevate serum LDH and MDA levels in mice. This is due to exercise-induced muscle cell damage and increased anaerobic metabolism. Electron microscopy results also revealed poor mitochondrial status in both the MC and Model groups. Mitochondria are associated with cellular energy metabolism, and impaired mitochondrial function can lead to increased reactive oxygen species (ROS), demonstrating that fatigue modeling in the Model group mice caused oxidative stress damage and dysregulation of cellular energy metabolism. Overall, fatigue induced by dietary restriction and exhaustive swimming results in poor recovery in mice, leading to slow weight restoration, mitochondrial damage, and even impacts on immune organs, aligning with the expected model outcomes.

IL-10 and TGF-β have dual roles in anti-inflammation and immune regulation. During disease recovery, they can limit inflammatory responses to promote tissue repair [29]. IL-6 [30] and TNF-α [31] are interactive inflammatory factors that elevate during the acute phase but may persist in the convalescence phase, causing tissue damage and fatigue [32]. The mild inflammatory response and elevated anti-inflammatory factors observed in the Model group mice indicate the cytokine status during the convalescence phase of the Model group mice.

Influenza is self-limiting and usually resolves within about a week. However, in cases of severe viral infection where the virus invades the lower respiratory tract, various symptoms can still persist during the recovery period, lasting weeks or longer [33,34]. The symptoms mainly manifest as fatigue, decreased immunity, and reduced work and exercise capacity, among others. The fatigue, immune suppression, oxidative stress, and slow weight gain observed in the Model group mice align with these symptoms. These symptoms share some similarities with PVFS. PVFS is also closely associated with mitochondrial dysfunction [35] and chronic inflammation [36] (such as elevated IL-6 and TNF-α levels). However, this animal model cannot be fully equated with PVFS. The diagnosis of PVFS requires persistent fatigue lasting six months or longer following a confirmed acute viral infection, accompanied by neuropsychiatric symptoms and somatic manifestations such as arthralgia [12,37], presenting a more complex clinical picture. Nevertheless, due to symptom similarities, the design methodology of the disease recovery phase and symptom superposition in this model still holds reference significance for PVFS animal models, including those for Long COVID or other viral pneumonia recovery phases.

However, this study still has some limitations. An inadequate control group setup constitutes the main flaw in the experimental design of this study. The use of oseltamivir serves as one of the variables in the modeling process, and the absence of a separate group using oseltamivir alone to treat IAV-infected mice prevents specific demonstration of oseltamivir’s effects on recovery-phase symptoms in this study, which represents a regrettable limitation. We plan to further investigate oseltamivir’s role in future research. Meanwhile, this study only analyzed three organs (lungs, spleen, thymus), failing to examine metabolic organs such as the liver, resulting in incomplete model analysis and a limited exploratory scope of the research.

## Figures and Tables

**Table 1:** Primer sequences for RT-qPCR.

Name	Sequence (5′–3′)
*GAPDH* (Forward primer) *GAPDH* (Reverse primer)	AGCTCGCTGTGAGCTGCTGAC TGTACACATGTATTCACGTCTG
*IAV NP* (Forward primer) *IAV NP* (Reverse primer)	CCTGTGTGTATGGACCTGCC CTCTTGGGACCACCTTCGTC
*IL-10* (Forward primer) *IL-10* (Reverse primer)	AGAGAAGCATGGCCCAGAAATCAAG AGAGAAGCATGGCCCAGAAATCAAG
*IL-6* (Forward primer) *IL-6* (Reverse primer)	TTCTTGGGACTGATGCTGGTGAC CTGTTGGGAGTGGTATCCTCTGTG
*TNF-α* (Forward primer) *TNF-α* (Reverse primer)	TAGCCCACGTCGTAGCAAAC ACAAGGTACAACCCATCGGC
*TGF-β* (Forward primer) *TGF-β* (Reverse primer)	AAGGAGACGGAATACAGGGC GGAAGGGCCGGTTCATGT

## Data Availability

All data are provided within the manuscript.

## Abbreviations

The following abbreviations are used in this manuscript:

IAV	Influenza A virus
PASC	SARS-CoV-2 infection
TCM	Traditional Chinese medicine
dpi	days post-inoculation
PVFS	post-viral fatigue syndrome
SHS	suboptimal health status
NP	Nucleoprotein
MDA	malondialdehyde
LDH	lactate dehydrogenase
SOD	total superoxide dismutase
